# Genetic Variation in *PADI6-PADI4* on 1p36.13 Is Associated with Common Forms of Human Generalized Epilepsy

**DOI:** 10.3390/genes12091441

**Published:** 2021-09-18

**Authors:** Russell J. Buono, Jonathan P. Bradfield, Zhi Wei, Michael R. Sperling, Dennis J. Dlugos, Michael D. Privitera, Jacqueline A. French, Warren Lo, Patrick Cossette, Steven C. Schachter, Heather Basehore, Falk W. Lohoff, Struan F. A. Grant, Thomas N. Ferraro, Hakon Hakonarson

**Affiliations:** 1 Department of Biomedical Sciences, Cooper Medical School of Rowan University, Camden, NJ 08103, USA; ferrarot@rowan.edu; 2 Center for Applied Genomics, The Children’s Hospital of Philadelphia, Philadelphia, PA 19104, USA; bradfield@email.chop.edu (J.P.B.); grants@email.chop.edu (S.F.A.G.); hakonarson@chop.edu (H.H.); 3 Department of Neurology, Thomas Jefferson University, Philadelphia, PA 19107, USA; michael.sperling@jefferson.edu; 4 Department of Computer Science, New Jersey Institute of Technology, Newark, NJ 07102, USA; zhiwei@njit.edu; 5 Department of Neurology and Pediatrics, The Children’s Hospital of Philadelphia, Philadelphia, PA 19104, USA; DLUGOS@email.chop.edu; 6 Department of Neurology, University of Cincinnati College of Medicine, Cincinnati, OH 45267, USA; michael.privitera@uc.edu; 7 Department of Neurology, New York University, New York, NY 10016, USA; Jacqueline.french@nyulangone.org; 8 Department of Neurology, Nationwide Children’s Hospital, Columbus, OH 43205, USA; warren.lo@nationwidechildrens.org; 9 Montreal University Health Center, University of Montreal, Montreal, QC H3C 3J7, Canada; patrick.cossette@umontreal.ca; 10 Departments of Neurology, Massachusetts General Hospital, Beth Israel Deaconess Medical Center, Harvard Medical School, Boston, MA 02114, USA; sschacht@bidmc.harvard.edu; 11 Research Service, Coatesville Veteran’s Affairs Medical Center, Coatesville, PA 19320, USA; Heather.Basehore@va.gov; 12 Section on Clinical Genomics and Experimental Therapeutics, NIAAA, NIH, Bethesda, MD 20892, USA; falk.lohoff@nih.gov

**Keywords:** epilepsy, human genetics, association study

## Abstract

We performed a genome-wide association study (GWAS) to identify genetic variation associated with common forms of idiopathic generalized epilepsy (GE) and focal epilepsy (FE). Using a cohort of 2220 patients and 14,448 controls, we searched for single nucleotide polymorphisms (SNPs) associated with GE, FE and both forms combined. We did not find any SNPs that reached genome-wide statistical significance (*p* ≤ 5 × 10^−8^) when comparing all cases to all controls, and few SNPs of interest comparing FE cases to controls. However, we document multiple linked SNPs in the *PADI6*-*PADI4* genes that reach genome-wide significance and are associated with disease when comparing GE cases alone to controls. *PADI* genes encode enzymes that deiminate arginine to citrulline in molecular pathways related to epigenetic regulation of histones and autoantibody formation. Although epilepsy genetics and treatment are focused strongly on ion channel and neurotransmitter mechanisms, these results suggest that epigenetic control of gene expression and the formation of autoantibodies may also play roles in epileptogenesis.

## 1. Introduction

Epilepsy is an umbrella term for a heterogeneous group of diseases that involve recurring seizures of many varieties as well as associated comorbidities. It is one of the most common of neurological illnesses, with a global incidence of 0.3–1.7% [1]. Although epilepsy incidence does not vary based on geography, culture or sex, it has nonetheless been difficult to estimate worldwide incidence due to socioeconomic variables between low- and high-income countries [2].

Some forms of epilepsy are rare, caused by a single gene mutation inherited in a predictable Mendelian pattern [3]. However, the vast majority of patients suffer from epilepsy subtypes of unknown etiology. These include both generalized epilepsies (GE, also called genetic generalized epilepsies: GGE) such as juvenile myoclonic epilepsy (JME) or childhood absence epilepsy (CAE), as well as focal epilepsies (FE) including temporal lobe epilepsy (TLE) with or without mesial temporal sclerosis (MTS). These common forms present as complex traits with no clear mode of inheritance, suggesting that they are caused by multiple genetic variants interacting with environmental influences [3]. Genome-wide association studies (GWAS) have been used to identify specific genetic variants that influence the etiology of many complex human traits and, in recent years, GWAS have successfully identified factors contributing to common illnesses [4]. Documenting an association between a common disease and common genetic variations (i.e., those occurring in >1% of chromosomes tested) opens new avenues of research into pathophysiological mechanisms.

To date, five epilepsy GWAS have been published. The first included cohorts of patients from Europe, USA and the UK who were diagnosed as having FE from both unknown and known causes, the latter including conditions such as infection, tumor or trauma [5]. No single nucleotide polymorphisms (SNPs) reached the conventionally accepted threshold for declaring genome-wide statistical significance (*p* < 5 × 10^−8^) [5]. There were several regions harboring SNPs that gave suggestive association (5 × 10^−8^ < *p* < 5 × 10^−6^) and it is likely that there were multiple false-negative (i.e., true-positive) loci represented within this level of statistical noise [4]. The second GWAS examined patients from China with FE, again including cases of both known and unknown cause [6]. In this study, only one variant, an SNP within *CAMSAP1L1* on 1q32, reached genome-wide significance (*p* = 1 × 10^−8^) [6]. The third epilepsy GWAS examined patients of European ancestry with idiopathic GE, including presumed genetic absence epilepsy (GAE), CAE, juvenile absence (JAE) and JME (EPICURE Consortium; EMINet Consortium) [7]. The patients were separated into two cohorts, one for discovery and one for replication, and a two-stage statistical analysis approach was employed. No SNPs reached genome-wide significance in the discovery cohort when GE, GAE or JME were compared individually to controls. However, when the replication cohort was processed and both datasets were combined by a meta-analysis, there were several chromosomes where markers reached genome-wide significance, including regions on 2p16.1 and 17q21.32 for GE, 2q22.3 for GAE and 1q43 for JME; in addition, there was a region of suggestive association at 2q24.3 for GE [7]. Next, a meta-analysis of prior GWAS [5,7], as well as the patients from this present study and other cohorts from different groups, was published under the auspices of the International League Against Epilepsy (ILAE) led by a Consortium on Complex Epilepsies [8]. This latter report included 8696 epilepsy patients and 26,157 controls and, compared to prior published work, it used a more stringent statistical criterion to declare genome-wide significance (*p* < 1.66 × 10^−8^). Results from the comparison between all patients with epilepsy and all control individuals revealed a number of genome-wide significant SNPs at 2q24.3 (*p* = 8.71 × 10^−10^), implicating *SCN1A* (a sodium ion channel subunit gene), and at 4p15.1 (*p* = 5.44 × 10^−9^), implicating *PCDH7* (a protocadherin gene). In the ILAE meta-analysis of the GE cohort, a single SNP at 2p16.1 (*p* = 9.99 × 10^−9^) was identified, most strongly implicating *VRK2* or *FANCL.* In addition, a statistical signal was detected at multiple SNPs in the *MMP8* locus on 11q22.2 (*p* < 2.37 × 10^−8^). The meta-analysis did not detect any SNPs that achieved genome-wide significance in the FE cohort. Most recently, the ILAE reported on an updated cohort that contained 15,212 patients and 29,677 controls, identifying 11 novel variations that reached genome-wide significance in the GE cohort and variants associated with all epilepsies, as well as with FE specifically [9].

Here, we report a GWAS on our cohort of patients we designate as the “Philadelphia Cohort”. The patients are all from North America and have common forms of GE and FE (see Materials and Methods). Our study results identify novel genetic loci that are potentially involved in the pathogenesis of epilepsy and that may provide entry points into novel pathways for possible therapeutic intervention [1].

## 2. Materials and Methods

### 2.1. Study Subjects

All subjects, including patients and controls, completed the informed consent process approved by local Institutional Review Boards at participating clinical sites. Peripheral blood samples were the preferred method for DNA sampling, but saliva samples were collected for some pediatric cases. Clinical inclusion criteria for GE and FE have been published previously [10]. These inclusion criteria give diagnostic age ranges for each of the epilepsy types collected and the cohorts have roughly equal distributions of male and female subjects. This cohort consisted of 2220 patients and 14,488 controls with 964 focal cases and 827 generalized cases collected directly by participating sites. Approximately 80% of the samples came from Jefferson, The Children’s Hospital of Philadelphia (CHOP) and Nationwide Children’s Hospital, with 20% coming from the remaining four sites (University of Pennsylvania, University of Cincinnati, University of Montreal and MGH/Harvard/Beth Israel Deaconess). Another 429 samples were added via screening of electronic health records from CHOP. Controls were also recruited at individual clinical sites with the majority (>95%) coming from the DNA repository at the Center for Applied Genomics (CAG) at CHOP. Case and controls were matched by ancestry and GWAS analysis used sex as a covariant.

### 2.2. Ethical Approval Code

All subjects gave their informed consent for inclusion before they participated in the study. The study was conducted in accordance with the Declaration of Helsinki, and the protocol was approved by the Ethics Committee of each participating institution under the overall auspices of the Institutional Review Board at Thomas Jefferson University Hospital (protocol #10F.208 approved 11 July 2019).

### 2.3. Overview: Genome-Wide Association Meta-Analysis

For gene discovery, we utilized cohorts in which the majority of cases were collected specifically for genetic studies and were recruited through epilepsy specialty physicians. We collected a smaller number of subjects via identification from the electronic medical record (EMR). To qualify for inclusion as a GE or FE case via EMR, an individual had to have an ICD9 code specifying that specific subtype of epilepsy (e.g., 345.1 or 345.4) and had to have been seen by a neurologist confirming the diagnosis (of GE or FE). All other ICD9 code diagnoses that pointed to idiopathic epilepsy were included in the ‘all epilepsy’ category. Table 1 presents information on patient ancestry, data acquisition chip used for genotyping and the type of epilepsy (GE or FE). For patients of European and African ancestries, we used METAL meta-analysis on cohorts 1, 3, and 5 and 2, 4 and 6, respectively [11]. All cases were matched with controls that were genotyped on the same platform with similar proportions of each cohort typed on each of the 3 platforms. For the combined European and African ancestry trans-ethnic meta-analysis, we utilized MANTRA and a Baysian approach [12].

### 2.4. Meta-Analysis: METAL and MANTRA

The genome-wide analyses of the European and Africa ancestry cohorts were meta-analyzed separately using METAL. MANTRA was then used for trans-ethnic meta-analysis. Sample numbers used are shown in Table 1. A total of 2220 epilepsy patient samples passed quality control and were included in the analysis. Of these, 964 were FE, 827 were GE and an additional 429 were from electronic records and not categorized as GE or FE. The control group consisted of 14,488 samples that passed all quality controls for genotyping. Statistical significance was set at genome-wide *p* ≤ 5 × 10^−8^ for METAL analysis. Statistical significance was set at Bayes Factor ≥ 6.00 for the MANTRA analysis.

### 2.5. Genotyping

We performed high-throughput genome-wide genotyping using three different high-density SNP arrays. Samples included in the discovery phase of the analysis were genotyped on the Illumina Infinium HumanHap550, Human610-Quad and HumanOmniExpress platforms.

### 2.6. Ancestry Determination

Data were pre-filtered for sample call rate > 95%, SNP call rate > 95% and minor allele frequency > 1%. The genotypes were then combined with those derived from overlapping SNPs within HapMap Phase 3. The Genome-wide Complex Trait Analysis (GCTA) program was used to determine the eigenvalues and eigenvectors of the samples [13]. The resulting top 10 eigenvectors were then used as input for a k-nearest neighbor‘s algorithm, which was trained on the Hapmap Phase 3 samples and their respective ancestries. The samples were then classified as the ancestry of the nearest Hapmap Phase 3 neighbor. The ‘knn’ function in the ‘class’ package in R was used for the k-nearest neighbor classification algorithm (https://stat.ethz.ch/R-manual/R-devel/library/class/html/knn.html) (accessed on 21 May 2017).

### 2.7. Imputation

Prior to imputation, samples were only retained in the analysis if they had either European or African ancestry, as these racial groups were the only ones of appropriate sample size. Samples were then grouped into their respective cohorts. Cohorts in which there were multiple BeadChip types were only analyzed with the subset of SNPs common to the different BeadChip types. Samples were then retained only if they had a sample call rate > 95% and were estimated to have an identity-by-descent proportion < 0.1875 with any other sample. SNPs were retained if they had a call rate > 95%, minor allele frequency > 1% and Hardy–Weinberg equilibrium (HWE) *p* > 1 × 10^−6^. All quality control was performed using PLINK [14]. All SNP positions are based on hg19 build. Samples were then pre-phased and genotypes imputed with the 1000 Genome phase 1 Interim haplotypes using Impute v2 [15]. Cases and controls were genotyped on different chip platforms, and all were matched to the appropriate chip and imputation data for final analyses (see Table 1).

### 2.8. SNP Validation

In order to test the accuracy of imputation, we selected a subset of cases and controls for Sanger sequencing using rs36067110 on 1p36.13 as a representative SNP. The forward sequencing primer was 5′-CTGTGCCTGGCCATATCATCT-3′ and the reverse sequencing primer was 5′-CCGTTGGTGGTAGGTGTCTAA-3′.

### 2.9. Genome-Wide Association

Prior to association analyses, SNPs were removed from the dataset if they had an info value < 0.8, minor allele frequency < 1% or HWE *p* < 1 × 10^−6^. All remaining SNPs were tested by logistic regression using the top 3 eigenvectors and sex as covariates for the 3 phenotypes. We used SNPtest for the association analysis (https://mathgen.stats.ox.ac.uk/genetics_software/snptest/snptest.html) (accessed on 21 May 2017).

### 2.10. Meta-Analysis

The cohorts separated by ancestry were meta-analyzed using METAL applying an inverse variance-based algorithm [15]. SNPs were removed from the meta-analysis if they had a heterogeneity (*p* < 0.05 or I^2^ < 50) or were missing from one of the groups for quality control reasons. Individual study- and meta-analysis-level genomic control was used prior to calculation of final ancestry-specific meta-analysis statistics. An SNP in the ancestry-specific meta-analysis was considered to be genome-wide significant with a *p*-value ≤ 5 × 10^−8^. Ancestry-level meta-analysis statistics were then combined using MANTRA in a trans-ethnic meta-analysis. The number of samples in each analysis was calculated as the effective sample size *N_eff_* = 4/(1/*N*_cases_ + 1/*N*_ctrls_). An SNP in the trans-ethnic meta-analysis was considered to be genome-wide significant with a log_10_ (Bayes Factor) ≥ 6.

## 3. Results

### 3.1. METAL and MANTRA for All Epilepsy Patients vs. All Controls

When comparing all patients to all controls, no SNPs reached the threshold for statistical significance in either METAL or MANTRA analyses. 

### 3.2. METAL for Patients of European Ancestry with GE

Table 2 shows that three SNPs reached genome-wide significance (*p* < 5 × 10^−8^) in the METAL analysis. The markers are located at 1p36.13 in the *PADI4-PADI6* locus. Furthermore, six additional SNPs in this region reached *p*-values of 5.3–8.1 × 10^−8^, resulting in nine linked markers with *p*-values between 10^−7^ and 10^−8^. These data document a positive association between variants in *PADI6*-*PADI4* and GE in European subjects.

### 3.3. METAL for GE in Patients of African Ancestry

Table 2 shows that no SNPs reached genome-wide significance when comparing patients of African ancestry with GE to controls. However, suggestive *p* values (~10^−5^) were found at three linked markers on the X chromosome near *TMEM47,* as was also found for patients of European ancestry.

### 3.4. MANTRA for GE Trans-Ethnic Analysis

Table 2 shows the results of the MANTRA analysis on all patients with GE. These data support the METAL analysis of European patients with GE, as the same three *PADI6* SNPs that reach genome-wide significance in the METAL analysis also reach genome-wide significance in the Bayes analysis, with Bayes Factors between 6.02 and 6.21. Two additional markers in *PADI4* reached Bayes Factors of 5.99 and 5.98. Four more SNPs in *PADI4* reached Bayes factors between 5.71 and 5.75. These data confirm a strong association between GE and *PADI4-PADI6*.

In the METAL analysis, the association between GE and SNPs on the X chromosome near *TMEM47* gave a *p*-value of 10^−5^ for patients of both European and African ancestry. Combined ancestry data with MANTRA show that two of these markers reach genome-wide significance (rs5928634, Bayes = 6.39 and rs5927306 Bayes = 6.23). One SNP on chromosome 17 reached a statistically significant Bayes Factor of 6.16 with a METAL *p*-value in Europeans of 3.98 × 10^−8^); however, this is a lone marker in a gene poor region and most likely represents a false-positive result.

### 3.5. METAL for FE Patients of European Ancestry

Table 3 shows the results of METAL analysis for the cohort of European FE patients. Only one marker, on the X chromosome (X-73537149) in *MAP2K4P1,* reached METAL significance in FE patients of European ancestry (*p* = 2.45 × 10^−8^). Once again, this lone marker, which is in a pseudo-gene, most likely represents a false-positive signal.

### 3.6. METAL for Patients of African Ancestry with FE

Table 3 shows the results of METAL analysis for the cohort of African patients with FE. Overall, four SNPs reached genome-wide significance; however, only two were linked and found in the same gene, *RANBP3*, on chromosome 19 (rs114591251, *p* = 3.66 × 10^−8^ and 19-5961503, *p* = 2.15 × 10^−8^). The other two are single markers, one in the *ASS1* gene on chromosome 9 and another in a gene poor region of chromosome 2 (rs12554609 in *ASS1, p* = 1.08 × 10^−9^, and rs58069848, *p* = 3.82 × 10^−8^).

### 3.7. MANTRA for FE Trans-Ethnic Analysis

Table 3 shows the results of the MANTRA trans-ethnic analyses for all patients with FE. Interestingly, for this cohort, the analysis calculated genome-wide significance for the single marker in *ASS1* noted above (Bayes 7.2) and the two linked SNPs in *RANBP3* also noted above (Bayes = 6.30 and 6.15, respectively). The lone marker in *MAP2K4P1* that reached genome-wide significance in the European FE METAL analysis is also statistically significant in MANTRA (Bayes = 6.29). The lone marker in the gene-poor region of chromosome 2 also shows a statistically significant MANTRA signal (Bayes = 6.13). In addition, the MANTRA results provide evidence for a suggestive association at 18q12.3 in the *PIK3C3* locus. Two SNPs in this region are very close to the threshold for genome-wide significance with Bayes factors at 5.99 and 5.96. Seven additional SNPs in this locus had Bayes factors between 5.65 and 5.94, and *p*-values in the African cohort via METAL that ranged from 1.2 × 10^−7^ to 5.1 × 10^−8^. These combined data provide evidence for a suggestive association between FE and *PIK3C3.* Further study of this locus is warranted, especially in patients of African ancestry.

### 3.8. SNP Validation

**Figure 1:**
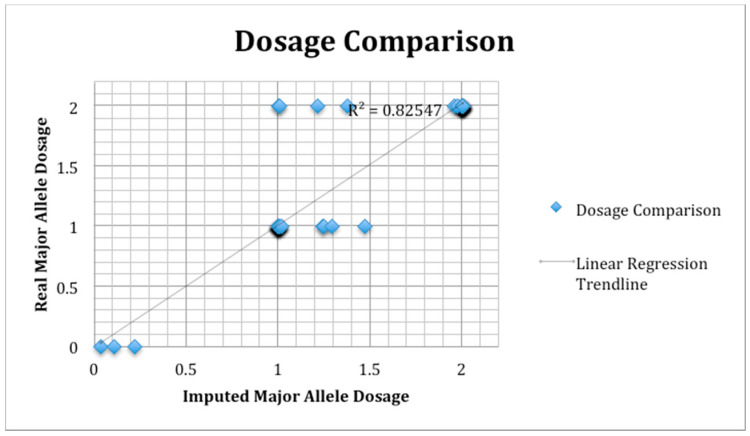
SNP validation by Sanger sequencing. Plot of Sanger sequence minor allele dosage vs. the imputed minor allele dosage. A set of 57 samples (n = 25 cases and n = 32 controls) was chosen for validation using rs36067110 on 1p36.13 as a representative SNP. The overall correlation coefficient of imputed SNP dosage vs. Sanger sequence SNP dosage was 0.826.

In order to test the accuracy of SNP imputation, we selected a subset of cases and controls for Sanger sequencing using rs36067110 on 1p36.13 as a representative locus. The specific subset of samples for validation was chosen based on case/control status and the imputed presence/absence of the respective alleles. We sequenced 57 samples (n = 25 cases and n = 32 controls), with two samples failing. A plot of the Sanger sequence minor allele dosage vs. the imputed minor allele dosage is shown in Figure 1. The overall correlation coefficient of imputed SNP dosage vs. Sanger sequence SNP dosage was 0.826.

### 3.9. Manhattan Plots

**Figure 2:**
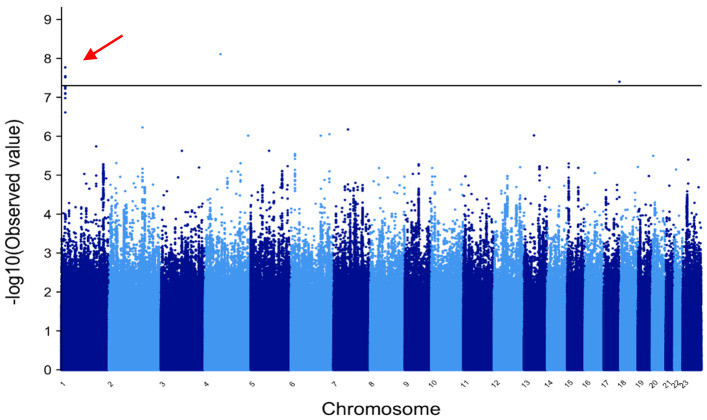
METAL Manhattan plot for generalized epilepsy European ancestry. Manhattan plots for the METAL analysis of generalized epilepsy patients of European ancestry. Multiple linked markers in the *PADI4-6* locus reached genome-wide statistical significance for patients of European ancestry. No markers reached genome-wide significance in patients of African ancestry (plot not shown).

Figure 2 shows the Manhattan plot for the METAL analysis of GE in patients of European ancestry. Note the three linked markers on chromosome 1 in the *PADI4-PADI6* locus that reach genome-wide statistical significance (red arrow) and an additional five linked markers in this region that are close to genome-wide significance.

**Figure 3:**
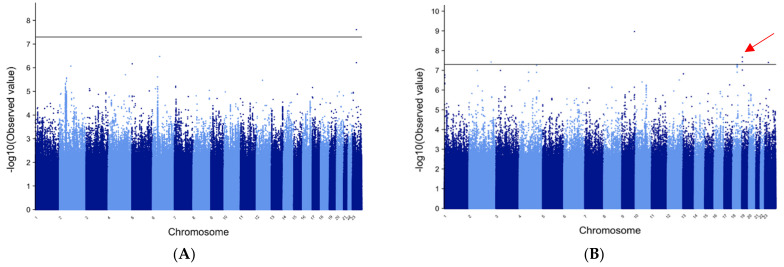
METAL Manhattan plots for focal epilepsy A. Manhattan plots for the METAL analysis of focal epilepsy patients: (**A**) patients of European ancestry; (**B**) patients of African ancestry. Few markers reach levels of statistical significance; however, 12 linked markers in *PIKC3* reach levels strongly suggestive of association in the patients of African ancestry (red arrow).

Figure 3 shows the Manhattan plots for the METAL analysis of FE in patients of both European and African ancestry. Of note is the solo marker in *MAP2K4P1* that reaches genome-wide statistical significance in the cohort of European patients and is most likely a false-positive signal. Similarly, in the African cohort, a single marker in *ASS1* and a single marker in a gene-poor region on the X chromosome near *NUDT10* reach genome-wide significance, but again, these likely represent false positives since no other linked marker shows significant or suggestive association. Two linked SNPs in *RANBP3* reached genome-wide significance in the African cohort. In addition, twelve linked SNPs in the *PIKC3* gene reached *p*-values of 10^−5^ in the METAL analysis in patients of African descent and two reached 5.99 and 5.96 Bayes factors in the MANTRA analysis.

**Figure 4:**
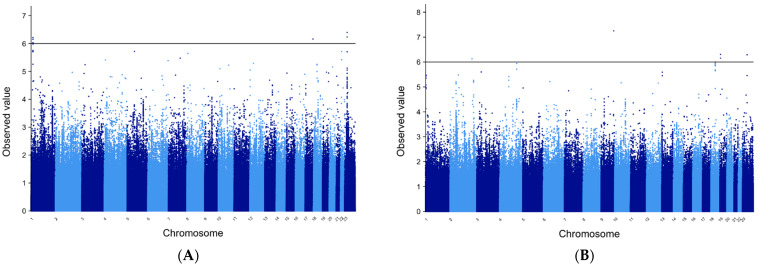
MANTRA Manhattan plots. Manhattan plots for the MANTRA trans-ethnic analysis (**A**) GE; (**B**) FE. Note markers in the *PADI4-PADI6* locus on 1p36 (above the horizontal line) that reach genome-wide levels of significance in patients with GE. Fewer positive results are observed in the FE cohort; however, two linked markers in *RANBP3* reach levels of statistical significance. This gene therefore warrants further study.

Figure 4. Manhattan plots for the trans-ethnic MANTRA analysis for both GE and FE, respectively. The *PADI4-PADI6* locus shows the strongest evidence for association in the combined GE cohorts. In addition, two linked markers on the X chromosome near the *TMEM47* gene reach the threshold for declaring statistical significance in the GE cohort and warrant further investigation. In the FE cohort, MANTRA results showed that two linked markers in *RANBP3* reach statistical significance along with solo markers in the *ASS1* and *MAP2K4P1* genes.

## 4. Discussion

We report positive results from a GWAS in common forms of epilepsy utilizing patients exclusively from the Philadelphia Cohort, samples that were included as part of a global (and ongoing) ILAE project [8,9]. In the present study, a set of nine linked markers in the *PADI4-PADI6* locus reach genome-wide or near genome-wide levels of statistical significance in patients with GE of European ancestry. Although this is a low yield, it is consistent with prior epilepsy GWAS using individual or a small number of patient cohorts [5,6,7].

Follow-up studies on larger patient populations assembled by combining cohorts from various international sites support the notion that, compared to FE, GE phenotypes have the strongest association with common genetic variation as a greater number of loci are detected with increasing sample size [8,9]. One possible explanation for the paucity of positive findings in FE is heterogeneity introduced by inclusion of cases with known symptomatic causes (arterio-venous malformation, tumor, infection or trauma), as examined in most prior studies [5,6,8,9]. These patients likely harbor genetic risk factors that are different than patients with idiopathic epilepsy and may dilute statistical power or introduce other confounds. Patients with FE in the Philadelphia Cohort were only included if they had no evidence for a symptomatic cause of their epilepsy.

Lack of replication of specific GWAS signals between independent cohorts remains a problem in the field of epilepsy research. Thus far, the *SCN1A* locus is the most replicated genetic risk factor found in epilepsy. It is a known causative factor in rare Mendelian epilepsies, as well as a risk factor in common epilepsies [8,9]. The PADI findings in our study are not reproduced at genome-wide significance levels in the larger meta-analyses performed thus far [8,9]. Heterogeneity of cohorts collected from different parts of the world likely contributes to this lack of replication at genome-wide levels. Thus, increasing sample size may not always increase power for specific signals, as important variations may be diluted when large heterogeneous populations are combined for analysis. In general, replication of SNP variations that reach genome-wide significance from prior published epilepsy GWAS have not been systematically studied in independent cohorts. While the ILAE continues to pool cohorts to create larger sample sizes for continued GWAS analysis, attempts to replicate prior findings in independent cohorts could help identify true positive findings. Replication could be conducted with less stringent requirements for claiming statistical significance, as correction for multiple testing is greatly reduced when specific markers are tested compared with a genome-wide analysis. It is possible that rare mutations in *PADI4* or *PADI6* are driving a synthetic association in our independent cohort as the *PADI* loci do not appear to be genome-wide significant in larger meta-analyses with other cohorts from around the world. Next generation sequencing of whole exomes from patients in our cohort would allow testing of the possibility that our reported results represent a synthetic association created by rare mutations in the Philadelphia Cohort. In every GWAS, there are a number of sub-threshold associations detected that point to genes participating in pathways that affect neurodevelopment or the electrical properties of neurons. Prioritizing this list for analysis based on biological plausibility, brain expression patterns and additional data linking genes to epilepsy phenotypes will identify additional candidate molecules that warrant further study.

Here, we report a genome-wide significant association between GE and a locus on chromosome 1 at 1p36.13 in the *PADI4*/*PADI6* gene region. Three SNP markers in this region reach genome-wide significance via METAL and six more SNPs have *p*-values between 2.4 × 10^−7^ and 5.3 × 10^−8^. These three SNPS also reach genome-wide significance in the MANTRA analysis (Bayes ≥ 6.00), as well as two additional markers at Bayes = 5.99 and 5.96. Of interest is the fact that deletion of regions on 1p36 in humans leads to variable clinical phenotypes, but the majority include epilepsy [16,17]. *PADI4-PADI6* is in the critical region of the 1p36 deletion syndromes; thus, our data point to this locus as potentially involved in the epilepsy phenotype in these individuals.

*PADI4* encodes an enzyme that mediates the conversion of arginine to citrulline as a post translational protein modification and is not able to convert free arginine to citrulline [18]. Genetic variation in *PADI4* has been associated with rheumatoid arthritis through the utilization of GWAS [18]. It has been proposed that increased citrullination of synovial proteins may alter their conformation and make them appear foreign, thereby triggering an autoimmune response [19]. In support of this hypothesis, overexpression of *PADI4* activity leads to loss in myelin in mouse nervous tissue, and citrullination of arginine residues in myelin proteins may induce autoantibody production [20,21]. More important is documentation that *PADI4* is expressed in both neurons and astrocytes of the hippocampus and cortex in human brain, and may contribute to formation of autoantibodies in Alzheimer’s disease [22]. Autoantibodies have been reported previously in patients with epilepsy, and over the past several decades, a substantial amount of literature on putative autoimmune defects in epilepsy has emerged [23]. It also is interesting to note that histone modifications include arginine-to-citrulline conversion [24], and this biochemical reaction decondenses chromatin and activates gene transcription, especially for genes that are expressed by early pluripotent stem cell progenitors [25]. Alternatively, arginine residues in histones are methylated as part of the epigenetic code, and conversion to citrulline would eliminate this site of epigenetic regulation. Thus, histone citrullination is an epigenetic process and it could be a mechanism that affects a wide variety of gene targets, including some that mediate epileptogenesis.

We also found suggestive evidence for an association between GE and markers on the X chromosome, with two markers near the *TMEM47* gene reaching significance via MANTRA (Bayes 6.39 and 6.23). *TMEM47* encodes a transmembrane protein of unknown function; thus, although the relationship to epileptogenesis is unclear, these results warrant further study.

In addition to positive results in GE, our data also provide suggestive evidence for several associations in FE, specifically in patients of African ancestry where we identified weak signals within *PIK3C3* and *RANBP3,* and weaker, single-marker signals in *ASS1* and *MAP2K4P1*. Although lone markers most likely represent false-positive results, *ASS1* is noteworthy in the context of our other findings because it encodes an enzyme that catalyzes the penultimate step in arginine synthesis and is upstream of the *PADI* enzymatic pathway. Nine linked markers with *p*-values just below genome-wide levels of significance in *PIK3C3* make this gene of particular interest. PIK genes participate in autophagy and lysosomal function [26]. Variations in *PIK3C3* have been associated with bipolar and schizophrenia in numerous studies [27]. The role of this gene in neurodevelopment is documented with a case report of a deletion in the gene in a patient with a specific learning disability [28].

## 5. Conclusions

We identified a locus at 1p36.13 that is associated strongly with common forms of human GE and, taken together, our data suggest that *PADI4* is the gene most likely to underlie this signal. We document multiple linked markers at genome-wide levels of significance by both METAL and MANTRA analyses. Additional markers of suggestive association warrant further study in separate cohorts. Future progress anticipated in larger epilepsy consortium projects will enable us to attempt replication of the results of the present study.

## Figures and Tables

**Table 1:** Epilepsy cohorts.

	Cohort 1	Cohort 2	Cohort 3	Cohort 4	Cohort 5	Cohort 6
Phenotyping	Physician verified	Physician verified	Physician verified	Physician verified	Electronic Medical Record	Electronic Medical Record
Genotyping Platform	550v1, 550v3, 610	550v1, 550v3, 610	OmniExpress	OmniExpress	550v1, 550v3, 610	550v1, 550v3, 610
Race	European American	African American	European American	African American	European American	African American
Patient categories/numbers analyzed						
All Epilepsy	819	159	483	106	432	221
Focal Epilepsy	378	92	287	61	91	55
Generalized Epilepsy	440	67	190	44	54	32
Controls	5736	2746	682	97	3443	1784

**Table 2:** METAL and MANTRA analyses for generalized epilepsy.

METAL							
		AFRICAN		EUROPEAN		MANTRA	
SNP	CHR	*p*-Value	β	*p*-Value	β	Bayes Factor	Gene
rs5928634	23	0.000005	0.75	0.00005	0.22	6.39 *	near TMEM47
rs5927306	23	0.000005	−0.75	0.00005	−0.22	6.23 *	near TMEM47
rs71644049	1	NA	NA	2.93 × 10^−8^ *	−1.49	6.21 *	PADI6 intron
17-77865302	17	NA	NA	3.98 × 10^−8^ *	1.36	6.16 *	none
rs71644048	1	NA	NA	3.05 × 10^−8^ *	−1.31	6.15 *	PADI6 intron
rs36067110	1	NA	NA	2.86 × 10^−8^ *	1.39	6.02 *	PADI6 intron
rs34199675	1	NA	NA	5.31 × 10^−8^	−1.33	5.99	PADI4 intron
rs34871124	1	NA	NA	5.41 × 10^−8^	−1.33	5.98	PADI4 intron
rs79100767	1	NA	NA	8.12 × 10^−8^	1.27	5.75	PADI4 intron
rs71644042	1	NA	NA	5.99 × 10^−8^	1.31	5.73	PADI4 intron
rs34018214	1	NA	NA	1.70 × 10^−8^ *	1.42	5.72	PADI4 intron
rs113755744	5	6.22 × 10^−8^	−2.55	NA	NA	5.72	none
rs79351721	22	8.30 × 10^−8^	−1.76	NA	NA	5.71	HPS4
rs4609339	23	0.00004	0.65	0.00005	0.22	5.71	near TMEM47
rs35240185	1	NA	NA	7.88 × 10^−8^	1.27	5.70	PADI4 syn
4-64069300	4	0.9	0.02	7.8 × 10^−9^	−1.97	4.80	near TECRL

* statistically significant for METAL (*p* ≤ 5 × 10^−8^) or MANTRA (Bayes ≥ 6.00). β represents the log of the odds ratio. A positive β is an odds ratio greater than 1 and represents a susceptibility allele. Conversely, a negative β is an odds ratio less than 1 and represents a resistance allele.

**Table 3:** METAL and MANTRA analyses for focal epilepsy.

METAL							
		AFRICAN		EUROPEAN		MANTRA	
SNP	CHR	*p*-Value	β	*p*-Value	β	Bayes Factor	Gene
rs12554609	9	1.08 × 10^−9^ *	−2.06	NA	NA	7.25 *	ASS1 intron
19-5961503	19	2.15 × 10^−8^ *	2.25	NA	NA	6.30 *	RANBP3
X-73537149	23	NA	NA	2.45 × 10^−8^ *	1.04	6.29 *	MAP2K4P1
rs114591251	19	3.66 × 10^−8^ *	−2.08	NA	NA	6.15 *	RANBP3
rs58069848	2	3.83 × 10^−8^ *	2.02	NA	NA	6.13 *	none
18-41994464	18	5.19 × 10^−8^	1.68	NA	NA	5.99	PIKC3
18-42005131	18	6.14 × 10^−8^	−1.67	NA	NA	5.95	PIKC3
rs116831532	18	5.11 × 10^−8^	1.68	NA	NA	0.95	PIKC3
18-42008808	18	6.97 × 10^−8^	1.66	NA	NA	5.95	PIKC3
rs12499431	4	5.68 × 10^−8^	2.30	NA	NA	5.94	SMARCA5
rs117507875	18	6.33 × 10^−8^	−1.67	NA	NA	5.89	PIKC3
rs114818774	18	6.93 × 10^−8^	−1.66	NA	NA	5.89	PIKC3
18-41998414	18	5.48 × 10^−8^	−1.68	NA	NA	5.88	PIKC3
18-42005556	18	6.20 × 10^−8^	−1.68	NA	NA	5.87	PIKC3
18-42004210	18	5.07 × 10^−8^	−1.68	NA	NA	5.87	PIKC3
rs114184892	18	6.55 × 10^−8^	−1.66	NA	NA	5.85	PIKC3
rs113055513	4	1.27 × 10^−7^	2.28	NA	NA	5.71	SMARCA5
18-41991735	18	5.09 × 10^−8^	1.68	NA	NA	5.68	PIKC3
18-42008321	18	1.24 × 10^−7^	1.62	NA	NA	5.65	PIKC3
X-51313775	X	4.03 × 10^−8^	1.05	NA	NA	4.67	near NUDT10

* statistically significant for METAL (*p* ≤ 5 × 10^−8^) or MANTRA (Bayes ≥ 6.00). β represents the log of the odds ratio. A positive β is an odds ratio greater than 1 and represents a susceptibility allele. Conversely, a negative β is an odds ratio less than 1 and represents a resistance allele.

## Data Availability

Summary statistics for this study are available upon request from the corresponding author RJB. The ILAE is depositing all summary statistics from this cohort and others around the world in dbGAP.
